# The physiology of survival: From the third man to placebo escape hatch?

**DOI:** 10.1113/EP094011

**Published:** 2026-07-31

**Authors:** Frank E. Marino, Alexander F. Marino

**Affiliations:** ^1^ School of Rural Medicine Charles Sturt University Orange New South Wales Australia; ^2^ School of Dentistry, Faculty of Health & Behavioural Sciences University of Queensland Brisbane Queensland Australia

## 1 THE THIRD MAN AND SURVIVAL AT THE PRECIPICE

During his 1916 Antarctic crossing of South Georgia, Shackleton later described the persistent sensation that his small group was not alone (Shackleton, 1919). Despite there being only three men present, each independently reported the feeling of a fourth companion travelling beside them. Shackleton himself reflected that during the crossing ‘it seemed to me often that we were four, not three’ (Geiger, 2009; Shackleton, 1919: p. 211). Similar experiences have since been described by mountaineers, sailors, polar explorers, soldiers, disaster survivors and endurance athletes operating under extreme fatigue, isolation, hypoxia, fear and environmental stress (Suedfeld & Mocellin, 1987). This phenomenon was later described as a ‘sensed presence’ (Suedfeld & Mocellin, 1987), noting that such experiences commonly emerge in extreme or unusual environments involving prolonged isolation, monotony, physical stress and environmental threat. In his compelling and classic text, John Geiger (2009), termed this phenomenon the ‘third man factor’, proposing that in conditions of extreme threat the human brain might generate a reassuring presence that sustains agency, hope and survival‐oriented behaviour.

Such accounts emerge near what have been described as ‘the precipice of thermoregulatory failure’, where survival depends upon the robustness of integrative physiological responses operating close to the limits of homeostasis (Kenney et al., 2004). Yet, physiology might be less about the passive encounter of biological limits and more about the active regulation of behaviour before those limits are even reached (Ulmer, 1996). Human tolerance to extreme heat, dehydration, hypoxia, exhaustion and environmental uncertainty is not determined simply by isolated physiological capacities, but by anticipatory systems that continuously adjust effort, pacing, perception and decision‐making to preserve survival (Marino, 2004).

From this perspective, the ‘third man’ might represent more than a perceptual distortion or cognitive suspension of disbelief. Importantly, and as previously argued, a sensed presence should not necessarily be viewed as psychopathology but might instead represent a normal adaptive response to unusual environments and that ‘Students of the presence phenomenon … usually fail to consider that it may be an adaptive response, a normal reaction to an abnormal situation’;a regulatory phenomenon that preserves agency, suppresses despair and maintains purposeful behaviour when the alternative might be behavioural withdrawal or homeostatic catastrophe. In such conditions, perception itself becomes physiologically relevant (Marcora & Staiano, 2010; Suedfeld & Mocellin, 1987: p. 49). The conjuring of a reassuring presence under extreme threat might itself represent an extension of survival physiology. The sensation that one is not alone or that survival remains possible might alter behaviour in ways that preserve movement, persistence and, ultimately, life itself.

Whether interpreted as neurophysiological adaptation, as predictive regulation or as an existential coping mechanism, the third man phenomenon raises a provocative physiological question; i.e., can expectation, belief and perceived support alter human function sufficiently to influence survival? At the precipice of human tolerance, the distinction between physiology and perception can become increasingly narrow. Survival rarely depends solely upon maximal physiological capacity; rather, the organism interprets threat, predicts tolerability and regulates behaviour under uncertainty.

## 2 PLACEBO: FROM ‘I SHALL PLEASE’ TO NEUROBIOLOGY

The conceptual ambiguity surrounding such phenomena can be traced back to the history of another familiar term, ‘placebo’, derived from the future tense of the Latin verb *placere* (‘to please’), translating literally to ‘I shall please’ (Shapiro, 1968). The usage of the term, however, originates from the Latin translation of Psalm 114:9 (modern translation Psalm 116:9), *Placebo Domino in regione vivorum* (‘I shall please the Lord in the land of the living’), and later became associated with the Office of the Dead during medieval funeral rites (Shapiro, 1968). Over time, the word acquired increasingly derisive connotations, referring to sycophantic ‘placebo singers’, who attended funerals to flatter the living and share in the hospitality that followed and for a free feed (De Craen et al., 1999). It was within this etymological setting that medicine later adopted the term placebo to describe treatments administered ‘more to please than benefit the patient’ (Shapiro, 1968). Thus, long before placebo entered the science of medicine, its usage was embodied with tensions between reality, perception, authenticity and ritual. Placebo rose to be a methodology to conduct randomized controlled trials and become a powerful explanatory construct within modern medical science (Kaptchuk, 1998).

This historical lineage has given placebo a powerful place in modern physiology and medicine. Contemporary placebo effects occupy a position where there is now substantial evidence that expectation and contextual belief can engage identifiable neurobiological pathways involving endogenous opioids, dopamine and autonomic regulation (Benedetti et al., 2005). For example, in Parkinson's disease, placebo administration has even been shown to induce measurable endogenous dopamine release within the striatum, suggesting that expectation alone might directly activate diseased physiological systems (de la Fuente‐Fernández et al., 2001).

## 3 EXPECTATION WITHOUT TREATMENT

The placebo effect can be regarded as responsible for genuine psychobiological responses arising from expectation, context and perceived support, but is also frequently invoked as a reason for a particular result, or a non‐result, implying illusion, deception or false attribution. In experimental science, placebo is simultaneously treated as a methodological control, a biological mechanism and, at times, a convenient explanation for findings that are subjective, weakly mechanistic or otherwise difficult to interpret. A further complication is whether placebo effects necessarily require deception. Traditionally, placebo has been associated with inert treatments administered in conditions where participants believe or at least thought they were receiving an active intervention (Nassif et al., 2014). However, many experimental protocols that alter physiological or behavioural responses involve no treatment at all, but rather manipulation of expectation, context or perceived reality.

In one of our earlier studies, cyclists who were deceived regarding the end‐point distance of a time trial subsequently altered pacing strategy and performance despite no change in physiology, equipment or pharmacological intervention (Paterson & Marino, 2004). Participants adjusted effort according to the expected demands of the task rather than the real demands imposed. Those who unknowingly completed a longer distance (36 km) subsequently increased power output and reduced completion time during a later 30 km trial, whereas those deceived into completing a shorter distance (24 km) reduced power output and completed the subsequent trial more slowly. Importantly, there was no inert substance, sham treatment or therapeutic procedure. Rather, performance regulation was altered solely by expectation and perceived task difficulty.

Similar findings have since emerged using a variety of deceptive protocols. Cyclists deceived into racing against an avatar that performed marginally above their previously established best performance improved subsequent 4 km time‐trial outcomes despite believing that they were competing against their true baseline effort (Stone et al., 2012). For some individuals, these performance gains persisted even after the deception had been disclosed, suggesting that the deceptive manipulation had altered the anticipated performance template itself (Paterson & Marino, 2004; Shei et al., 2016). Likewise, deceptive visual feedback involving clocks running at manipulated speeds, altered end‐point information or covertly augmented performance feedback has been shown to alter time to exhaustion, pacing strategy and power output (Castle et al., 2012; Stone et al., 2012).

Perhaps even more striking are studies manipulating thermal perception which demonstrated that cyclists exercising in hot, humid conditions improved self‐paced performance when deceptively informed that ambient and core temperatures were lower than their real values (Castle et al., 2012). The improvement occurred despite no difference in real core temperature response, suggesting that altered perception of thermal strain influenced behavioural regulation and pacing. Such findings resonate strongly with broader concepts of anticipatory regulation and survival physiology. Human beings rarely respond purely to physiological state alone; rather, behaviour is shaped continuously by perceived threat, expected effort and predicted tolerability. This broader interpretation is consistent with emerging placebo frameworks proposing that placebo effects are not confined to medicine but represent general interactions between the organism and its environment, in which expectation, conditioning, motivation and contextual meaning influence physiological regulation and physical performance (Pollo et al., 2011).

Collectively, these findings raise an important conceptual question: if belief alters physiological regulation and behaviour in the absence of any ‘treatment’, is this still placebo? Or does placebo merely represent one subset of a broader anticipatory physiology in which expectation, prediction and contextual meaning shape human function? This distinction matters because the term placebo is on occasion used imprecisely. In some contexts, placebo refers to deception; in others expectation; and in yet others conditioning, contextual meaning or therapeutic intervention. These mechanisms are not identical. A deceptive intervention may generate expectancy, but expectancy can also emerge without deception. Likewise, ritual, trust, prior experience and environmental cues can alter symptoms and behaviour independently of any inert treatment. Thus, placebo might be less a singular mechanism than it is a collection of expectation‐related processes that remain incompletely defined.

## 4 THE PLACEBO ESCAPE HATCH

The converse phenomenon, *nocebo*, further illustrates that expectation‐based regulation is not exclusively beneficial. Negative expectations can worsen symptoms, increase perceived pain, reduce treatment efficacy and even generate adverse effects in the absence of direct physiological insult (Watanabe et al., 2022). This has been recognized increasingly across multiple medical disciplines, including dentistry, where anxiety, clinician language, prior experience, catastrophizing and patient expectation can substantially shape pain perception and treatment outcomes. Experimental studies demonstrate that negative verbal suggestion can amplify pain, block analgesic responses and alter symptom experience despite identical physiological interventions. Emerging evidence further suggests that such effects can be socially transmitted, with parental anxiety and catastrophic framing influencing pain expectation, anxiety and symptom perception in children undergoing treatment (Schmidt et al., 2026). In this sense, placebo and nocebo might represent opposing manifestations of the same anticipatory regulatory architecture, shaped not only by physiology itself, but by predicted state, anticipated threat and interpersonal context.

The tendency to invoke placebo as a residual explanation is not new. Beecher's influential 1955 paper, *The Powerful Placebo*, helped to establish placebo as an explanatory construct in modern medicine (Beecher, 1955). It has been argued that the rise of placebo‐controlled trials transformed placebo into an almost paradoxical explanatory entity capable of mimicking potent therapies despite conceptual instability (Kaptchuk, 1998). Subsequent methodological analyses have suggested that placebo responses might reflect multiple overlapping phenomena, including regression to the mean, Hawthorne effects, expectancy, contextual meaning and true psychobiological placebo effects (Hawkins & Scott, 2010). Consequently, placebo is often treated as a singular mechanism despite being likely to represent several distinct regulatory and interpretive processes.

The possibility that expectation and belief influence physiological regulation should not be surprising. Human survival has always depended not merely on passive responses to the environment, but on anticipatory regulation under uncertainty. Perceptions of safety, threat, control and support influence behaviour, pacing, effort, pain tolerance and decision‐making long before physiological catastrophe occurs. From this perspective, placebo effects might represent a less dramatic but experimentally accessible manifestation of a broader survival physiology, in which prediction and perception shape human function.

The challenge, however, is that placebo has increasingly become a scientific escape hatch. Statements such as ‘the effect was likely placebo’ or ‘participants simply believed the intervention worked’ are often made without direct assessment of expectancy, blinding integrity, prior exposure, contextual cues or participant belief. In such cases, placebo risks becoming less a mechanism to be investigated and more a term used to avoid discussion rather than stimulate mechanistic inquiry.

## 5 EXPECTATION AS REGULATION

Finally, an unresolved tension remains regarding whether placebo methodologies are ethically justified simply because we might find them familiar. In clinical trials, placebo controls might require the withholding of established therapies in order that we might demonstrate efficacy beyond expectation and contextual effects (Annoni, 2018). Experimental protocols using deception deliberately manipulate belief and perception to examine physiological regulation. Although methodologically powerful, they remain ethically uncomfortable because they depend, in part, upon altering perceived reality of those under investigation or treatment. For if expectation itself is biologically active, then placebo and deception are not merely methodological tools; they become interventions that modify how physiology is regulated through perception, expectation and perceived survivability.

Perhaps the enduring significance of the third man phenomenon is not whether the sensed presence was ‘real’, but that it might reveal something fundamental about the biology of survival itself. Organisms surviving at the precipice of tolerance do not simply encounter physiological limits; they regulate behaviour, perception and meaning in ways that preserve function and postpone cellular catastrophe. Placebo effects might represent a less dramatic but experimentally accessible expression of that principle. The task for physiology is, therefore, not merely to determine whether placebo effects are ‘real’, but to understand how expectation, prediction and belief become integrated into the regulation of human behaviour and survival.

## AUTHOR CONTRIBUTIONS

Each author has read and approved the final version of this manuscript and agrees to be accountable for all aspects of the work. Each person designated as an author qualifies for authorship.

## CONFLICT OF INTEREST

The authors declare they have no conflict of interest.

## GENERATIVE AI STATEMENT

The authors confirm that no artificial intelligence tools, including large language models (LLMs), were used in the drafting or revision of this manuscript. All content was conceived, written and approved solely by the authors.
